# Effect of gestational age at birth on neonatal outcomes in gastroschisis^☆^

**DOI:** 10.1016/j.jpedsurg.2016.02.013

**Published:** 2016-05

**Authors:** Helen Carnaghan, David Baud, Eveline Lapidus-Krol, Greg Ryan, Prakesh S. Shah, Agostino Pierro, Simon Eaton

**Affiliations:** a UCL Institute of Child Health and Great Ormond Street Hospital for Children, London, UK; b Maternal-Fetal Medicine Unit, Mount Sinai Hospital, Toronto, Ontario, Canada; c Division of General and Thoracic Surgery, The Hospital for Sick Children, Toronto, Ontario, Canada; d Department of Pediatrics, Mount Sinai Hospital, Toronto, Ontario, Canada; e Materno-fetal and Obstetrics Research Unit, Department of Obstetrics and Gynecology, Maternity, University Hospital, Lausanne, Switzerland

**Keywords:** ENT, Time to full enteral feeds, LOS, Length of hospital stay, GA, Gestational age, PN, Parenteral nutrition, Gastroschisis, Gestational age, Preterm delivery, Neonatal outcome, Postnatal outcome, Enteral feeding

## Abstract

**Introduction:**

Induced birth of fetuses with gastroschisis from 34 weeks gestational age (GA) has been proposed to reduce bowel damage. We aimed to determine the effect of birth timing on time to full enteral feeds (ENT), length of hospital stay (LOS), and sepsis.

**Methods:**

A retrospective analysis (2000–2014) of gastroschisis born at ≥ 34 weeks GA was performed. Associations between birth timing and outcomes were analyzed by Mann–Whitney test, Cox regression, and Fisher's exact test.

**Results:**

217 patients were analyzed. Although there was no difference in ENT between those born at 34–36 + 6 weeks GA (median 28 range [6–639] days) compared with ≥ 37 weeks GA (27 [8–349] days) when analyzed by Mann–Whitney test (p = 0.5), Cox regression analysis revealed that lower birth GA significantly prolonged ENT (p = 0.001). LOS was significantly longer in those born at 34–36 + 6 weeks GA (42 [8–346] days) compared with ≥ 37 weeks GA 34 [11–349] days by both Mann–Whitney (p = 0.02) and Cox regression analysis (p < 0.0005). Incidence of sepsis was higher in infants born at 34–36 + 6 weeks (32%) vs. infants born at ≥ 37 weeks (17%; p = 0.02).

**Conclusions:**

Early birth of fetuses with gastroschisis was associated with delay in reaching full enteral feeds, prolonged hospitalization, and a higher incidence of sepsis.

Gastroschisis neonates are frequently affected by varying degrees of gut dysfunction with 49% of neonates requiring > 28 days of parenteral nutrition [1], [2]. However, the reasons for this gut dysfunction remain unknown and unlike other antenatally detected conditions, there are neither definitive antenatal findings that predict neonatal outcomes nor a robust in-utero treatment option that could improve neonatal gut function.

Elective birth at 37 to 38 weeks gestational age (GA) has been suggested following report of in-utero fetal deaths in late term pregnancies [3] and is practiced by many centers worldwide [4]. Some recent publications suggested that early term birth at around 37 weeks GA was associated with improved neonatal morbidity and mortality compared with birth at late term [5], [6], [7]. Furthermore, it has also been hypothesized that longer exposure to amniotic fluid has a deleterious effect on the developing bowel resulting in postnatal dysfunction [8], [9]. Therefore, some surgeons suggest that routine birth in the late preterm period between 34 and 36 + 6 weeks GA of otherwise healthy gastroschisis fetuses may improve neonatal gut function as a result of reduced bowel exposure to amniotic fluid.

The literature with regards to delivering gastroschisis pregnancies electively between 34 and 36 + 6 weeks to protect bowel from ongoing damage is conflicting with some studies in favor [10], [11], [12] and others against [4], [13], [14], [15]. A definitive randomized control study with enough power to answer this question is elusive owing to the relatively small numbers of gastroschisis neonates seen per centre per year. Our objective in this retrospective cohort study was to assess the association of timing of birth in fetuses with gastroschisis and time to full enteral feeds (ENT), length of stay (LOS) and sepsis.

## 1 Material and methods

We performed a retrospective study of gastroschisis neonates born at ≥ 34 weeks GA managed antenatally at Mount Sinai Hospital, Toronto and stabilized immediately after birth at Mount Sinai Hospital and managed postnatally at the Hospital for Sick Children, Toronto between January 2000 and July 2014. Any fetus or neonate treated outside of either center was excluded from the study. Approval was granted by the Institutional Research Ethics Boards of both hospitals to collect combined antenatal and neonatal data by retrospective chart review. Gastroschisis cases were identified through interrogation of the Mount Sinai and Hospital for Sick Children databases. Fetal and neonatal data were obtained from computerized charts at the respective hospitals.

During the study period pregnancies were monitored in Mount Sinai antenatal clinic at 4-weekly intervals until 30 weeks GA, 2-weekly intervals between 30 and 34 weeks, once-weekly after 34 weeks and twice-weekly after 35 weeks. Women were electively delivered vaginally by induction of labor at 37 weeks GA unless obstetric concerns necessitated conversion to cesarean section. Earlier birth was performed in the presence of nonreassuring biophysiological profile or nonstress testing, occasionally owing to presence of bowel dilatation, maternal health concerns or as a result of spontaneous labor. Emergent delivery was defined as being within 24 h of the examination that triggered concern, and semielective delivery as planned induction brought forward from original planned date, but at least 24 h later than the examination. Gestational age was estimated based on best obstetric estimate on early ultrasound, obstetric history and examination followed by pediatric estimate in that order.

The outcome measures were:

a. Time to full enteral feeds (ENT) defined as time taken from birth to full enteral feeds as described in the hospital notes;
b. Length of hospital stay (LOS) defined as time from birth to discharge or death;
c. Sepsis defined as isolation of a pathogenic organism from blood.

### 1.1 Statistical analysis

Neonates were divided in two groups: Those born between 34 and 36 + 6 weeks gestation and those born at or after 37 weeks gestation. Baseline comparison between two groups was conducted using Fisher's exact test. Outcomes were compared between two groups, with and without taking into account corrected GA. Mann Whitney, Cox regression analysis, and Fisher's exact test were utilized and data are presented as median [range]. Statistical analysis was performed using GraphPad Prism v6. and SPSS v22 software. A p value of < 0.05 was considered statistically significant.

## 2 Results

**Fig. 1:**
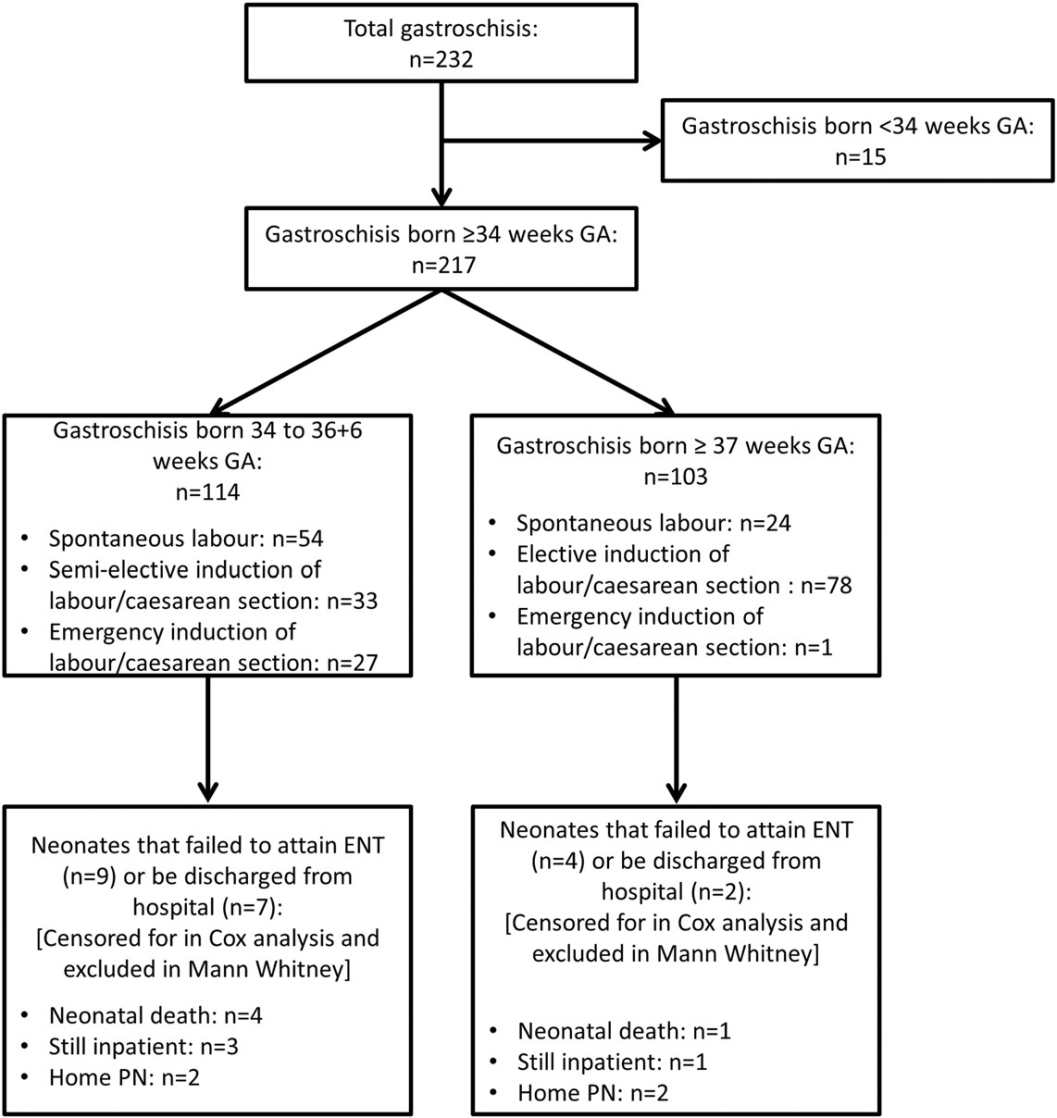
Flow diagram illustrating inclusion and exclusion of gastroschisis neonates.

A total of 232 gastroschisis neonates were born during the study period, of whom 15 were born at < 34 weeks and excluded from the study. Of the 217 neonates born at ≥ 34 weeks GA, 30 were complex including 14 who had associated atresia, 6 who had stenosis, 4 who had perforation and 15 who had necrosis (6 patients had two or more concomitant bowel pathologies). Five neonates died for the following reasons; 1 patient owing to necrotic bowel and multiorgan failure, 1 owing to necrotizing enterocolitis, 2 owing to parenteral nutrition associated liver failure and 1 owing to overwhelming sepsis. At the time of data collection, 4 complex patients were still increasing on feed volumes in hospital (3 necrosis and 1 atresia patient). A further 4 complex patients require home PN owing to short bowel syndrome (3 born with necrosis, 1 atresia). These 217 neonates were divided into two groups: those born at 34 to 36 + 6 weeks GA (n = 114) and those born at ≥ 37 weeks GA (n = 103, Fig. 1). There was a similar proportion of complex patients in the two groups (16% [n = 18] and 12% [n = 12] respectively [p = 0.43]). Table 1 delineates the antenatal concerns and reasons for preterm birth in those neonates born between 34 and 36 + 6 weeks gestation. Labor onset in the ≥ 37 weeks GA group included spontaneous in 24 patients, planned induction of labor/caesarean section in 78 and emergency caesarean section in 1.

Analysis of time to achieve ENT and LOS showed that there was no difference in ENT between those born at 34 to 36 + 6 weeks GA (median 28 range [6–639] days) compared with neonates born at ≥ 37 weeks GA (27 [8–349] days), p = 0.5. However, LOS was significantly longer in those born at 34 to 36 + 6 weeks GA (42 [8–346] days), compared with neonates born at ≥ 37 weeks GA (LOS 34 [11–349] days), p = 0.02 (Mann–Whitney excluding those patients not reaching full feeds or discharged from hospital, as appropriate). In order to account for the effect of prematurity, we analyzed corrected GA at ENT and LOS. The corrected GA at ENT was significantly lower in neonates born at 34 to 36 + 6 weeks GA (ENT 40 [36–125]) compared with those born ≥ 37 weeks GA (ENT 41[39–87] weeks, p = 0.009). However, corrected GA at discharge from hospital was similar for neonates born at 34 to 36 + 6 weeks GA (42 [37–84] weeks) compared with those born ≥ 37 weeks GA (44 [39–87] weeks, p = 0.36). Additionally, the proportion of infants having at least one episode of sepsis was significantly higher in infants born at 34 to 36 + 6 weeks GA (36/114, 32%) than those born at ≥ 37 weeks GA (18/103, 17%, p = 0.019).

**Fig. 2:**
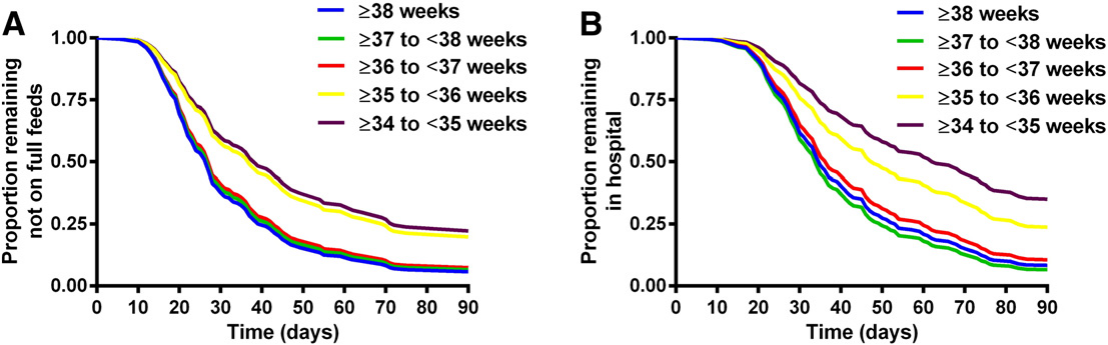
Effect of gestational age category on time to (A) full enteral feeds and (B) hospital discharge. Cox regression analyses of the entire cohort, adjusting for complex gastroschisis and gestational age category, and censoring for death, not achieving full feeds or not being discharged from hospital as appropriate.

To determine the effect of GA on ENT and LOS across the entire cohort we performed a Cox regression analysis, adjusting for complexity and GA and censoring for death, not achieving full feeds or not being discharged from hospital as appropriate. As expected, complexity at birth significantly prolonged ENT (hazard ratio for reaching full feeds 0.27 [95% CI: 0.17–0.44], p < 0.0005) and LOS (hazard ratio for hospital discharge 0.35 95% CI: [0.22–0.55], p < 0.0005) compared with simple gastroschisis. Birth at lower GA was associated with a significantly prolonged ENT (hazard ratio for reaching full feeds 0.79 per week earlier birth [95% CI: 0.69–0.90], p = 0.001) and LOS (hazard ratio for hospital discharge 0.75 per week earlier birth [95% CI: 0.66–0.86], p < 0.0005). In order to illustrate the effects of lower birth GA on ENT and LOS, we divided the patients into categories of birth GA and repeated the Cox regression analysis with the categorical GA variable. Cox regression curves are shown for each GA category for ENT (Fig. 2A) and LOS (Fig. 2B), although it must be emphasized that the analysis was only performed with GA categories in order to generate interpretable graphs, as the original regression analyses with birth GA as a continuous variable are more statistically powerful.

As many of the neonates born before 37 weeks were delivered for obstetric reasons, rather than planned early induction of labor, we also repeated the analysis for those neonates born at 34 to 36 + 6 weeks GA to examine the effect of mode of labor onset, although it should be noted that this is a post hoc analysis that is lacking in statistical power owing to small patient numbers in some of the labor mode categories. We compared spontaneous labor (5/54 complex patients, mean GA at birth 35.8 ± 0.1 weeks) with: (i) semielective early induction of labor/cesarean section (4/33 complex patients, mean GA at birth 36.1 ± 0.1 weeks); and (ii) emergency (3/27 complex patients, mean GA at birth 35.6 ± 0.2 weeks). The effect of lower GA at birth remained significant for ENT (hazard ratio for reaching full feeds 0.63 per week earlier birth [95% CI: 0.46–0.86], p = 0.004) and LOS (hazard ratio for hospital discharge 0.53 per week earlier birth [95% CI: 0.37–0.75], p < 0.0005). However, there was no significant effect of birth mode on ENT (hazard ratio 1.0 [0.6–1.7] p = 0.9 for nonurgent compared with spontaneous, hazard ratio 1.0 [0.6–1.8] p = 0.90 for emergency), or on LOS (hazard ratio 1.2 [0.7–2.0] p = 0.62 for nonurgent IOL, odds ratio 1.0 [0.6–1.9] p = 0.95 for emergency). We also analyzed the effect of labor onset mode in patients born at ≥ 37 weeks GA comparing spontaneous labor (2/24 complex patients, mean GA at birth 37.6 ± 0.18 weeks) with planned induction of labor/caesarean section (9/78 complex patients, mean GA at birth 37.4 ± 0.05 weeks); there was only one patient with emergent caesarean section in this GA group. There was no effect of planned induction/caesarean section compared with spontaneous labor on ENT (hazard ratio 0.6 [95% CI: 0.4–1.0], p = 0.06) or LOS (hazard ratio 0.7 [95% CI: 0.4–1.2], p = 0.17). Within the ≥ 37 weeks group, there was no effect of lower GA at birth on ENT (hazard ratio per week 0.9 [95% CI: 0.6–1.3], p = 0.5)) or LOS (hazard ratio per week 0.9 [95% CI: 0.6–1.4], p = 0.78).

## 3 Discussion

This large single center cohort shows a significant association between early GA at birth and prolonged ENT, LOS and increased risk of sepsis.

Analysis of the corrected gestational age at time to full feeds would suggest that delivery earlier than 37 weeks improves neonatal gut function. However, this is not owing to a faster time from birth to full enteral feeds, as time to full feeds is similar between those born before 37 weeks and those born after 37 weeks. Overall, younger GA in these gastroschisis infants is associated with a longer time postnatally to achieve full enteral feeds, as indicated by Cox regression analysis. As this cohort only includes gastroschisis infants, we are unable to determine whether this effect of younger GA on prolonging enteral feeds is specific to gastroschisis or is a general effect of prematurity. Although corrected GA at full enteral feeds is lower in the group born at less than 37 weeks, these infants are discharged from hospital at a similar corrected GA to those born at greater than 37 weeks, and therefore spend longer in hospital before discharge, as indicated by the Cox regression analysis of length of hospital stay. The longer time before achieving full feeds, and the longer time in hospital, place infants born before 37 weeks at increased risk of sepsis, as indicated by the nearly doubled (32% vs. 17%) proportion of infants having an episode of blood-culture positive sepsis in those born at less than 37 weeks.

We must acknowledge limitations of our study. Our study was a retrospective analysis including patients delivered at < 37 weeks GA on an emergent or semiurgent basis owing to maternal–fetal concerns, patients delivered at < 37 weeks owing to spontaneous labor and patients delivered at ≥ 37 weeks GA as per standard induction. Such comparisons could be affected by the overall health of the neonates with those delivered early for maternal or fetal concerns possibly having poorer outcomes as a result. We have tried to control for this in post hoc analyses adjusting for reason for early delivery, in which mode of labor onset did not significantly affect neonatal outcomes; however, we cannot be certain regarding the reasoning behind the decision to give birth in some women at 34 to 36 + 6 weeks while allowing others to progress to 37 weeks GA. There may have been additional confounders that we were unable to take account of. We are also unable to recommend elective delivery at approximately 37 weeks rather than later spontaneous delivery or induction of labor after 37 weeks. The effect of lower birth GA on ENT and LOS is consistent with other large [4], [16] and smaller [13], [17], [18] retrospective studies. Our findings indicate that birth in the term period (≥ 37 weeks GA) as opposed to the late preterm period (34 to 36 + 6 weeks GA) of otherwise healthy gastroschisis fetuses may be associated with improved outcomes, especially reduction of sepsis. Most centers advocate induction of labor in the early term period at around 37 weeks. This study was not designed to determine antenatal findings that would identify gastroschisis patients with the worst neonatal outcomes. Therefore, timing of birth for gastroschisis fetuses with evidence of fetal or maternal compromise should continue to be dictated by obstetric necessity rather than any specified GA until further evidence is available to guide birth timing for fetuses with gastroschisis.

## 4 Conclusion

Early birth of otherwise healthy gastroschisis fetuses did not appear to reduce bowel damage or improve bowel function and was not associated with differences in corrected postnatal age at which patients reach full feeds or are discharged from hospital.

## Figures and Tables

**Table 1:** Antenatal concerns and reasons for early birth in neonates born at 34 to 36 + 6 weeks GA.

Reason for early delivery/antenatal concerns	34–36 + 6 weeks GA spontaneous labor (n = 54)	34–36 + 6 weeks GA semielective early induction of labor (n = 33)	34–36 + 6 weeks GA emergency delivery (n = 27)
No antenatal concerns	39	6	0
Maternal factors			
Maternal alcohol/smoking/drug use	8	0	0
Maternal health reasons	0	2	1
Maternal infection e.g. pyelonephritis	1	0	0
Prolonged rupture of membranes	0	2	0
Fetal factors			
Bowel dilatation	4	10	6
Intrauterine growth restriction	0	3	1
Reduced fetal movements	1	1	8
Nonreassuring fetal heart rate	0	4	4
Nonreassuring biophysiological profile	1	3	5
Oligohydramnios	0	0	2
Unknown	0	2	0
